# Comprehensive identification of *NLP* gene family in *Panax ginseng*:*PgNLP02–01* and *PgNLP07* genes expression analysis in ginseng adventitious roots under MeJA response

**DOI:** 10.3389/fpls.2026.1927551

**Published:** 2026-08-18

**Authors:** Aimin Wang, Xinyue Hu, Meiyan Fan, Mingzhu Zhao, Yi Wang, Kangyu Wang, Meiping Zhang

**Affiliations:** 1 College of Life Science, Jilin Agricultural University, Changchun, Jilin, China; 2 Jilin Engineering Research Center Ginseng Genetic Resources Development and Utilization, Jilin Agricultural University, Changchun, Jilin, China

**Keywords:** functional gene mining, ginsenoside biosynthesis, *Panax ginseng*, *PgNLP* gene family, transcription factors

## Abstract

*Panax ginseng* is a perennial plant of the *Araliaceae* family. Ginsenosides in ginseng are important secondary metabolites with extremely high economic value. The *NLP* family constitutes an important class of transcription factors in plants that participate in regulating plant growth, development, secondary metabolism, and other biological processes. In this study, we identified 20 members of the *NLP* gene family in ginseng. Analyses were performed on gene structure, chromosomal localization and collinearity relationships, phylogenetic relationships, expression patterns, and cis-acting elements. Three candidate genes were finally screened out through SNP/InDel association analysis with ginsenosides, correlation analysis between gene expression levels and ginsenoside contents, and co-expression analysis with key enzyme genes. The expression profiles of these three candidate genes under MeJA induction were investigated, and their correlations with ginsenoside content under the same treatment were further analyzed. Consequently, two genes (*PgNLP02–01* and *PgNLP07*) associated with the regulation of ginsenoside biosynthesis were identified in this study. Co-expression trend analysis with 15 key enzyme genes revealed that the two genes negatively and positively regulated the expression of *CYP716A53v2_1* and *DS_1*, respectively, thereby clarifying the potential regulatory mechanism underlying ginsenoside biosynthesis in ginseng.

## 1 Introduction

Ginseng is a perennial herbaceous plant belonging to the genus *Panax* in the *Araliaceae* family and has a long medicinal history in China (Hu, 1976). It is mainly used for replenishing qi and rescuing collapse, nourishing the heart and calming the mind, tonifying the lung and relieving asthma, and invigorating the spleen and stopping diarrhea. Ginsenosides are a group of triterpenoid compounds in ginseng with extremely high medicinal and economic value, which are mainly divided into dammarane-and oleanolic acid-type (Ru et al., 2015). At present, most biosynthetic pathways of ginsenosides have been elucidated, and the key enzyme genes involved in these pathways have been continuously cloned and functionally verified (Lee et al., 2004; Kim et al., 2011; Kim et al., 2014; Han et al., 2020). However, ginsenoside biosynthesis is not a simple catalytic reaction, and various transcription factors are involved in regulating ginsenoside biosynthesis (Jang et al., 2024; Jiang et al., 2024; Jiang et al., 2024).

NLP (NIN-like protein) transcription factors represent a transcription factor family that is widely distributed from algae to higher plants (Bowman et al., 2017). Studies have demonstrated that the domains of NLP transcription factors are evolutionarily ancient and existed prior to the divergence of Viridiplantae (Schauser et al., 2005). This family contains conserved RWP-RK and PB1 domains (Chardin et al., 2014). The RWP-RK domain is responsible for DNA binding and executes biological functions in various biological processes (Konishi and Yanagisawa, 2013), whereas the PB1 domain mediates the formation of homodimers and heterodimers to regulate transcriptional activity (Konishi and Yanagisawa, 2019). Studies on plant NLP transcription factors have revealed that this family possesses diverse biological functions and plays crucial roles in plant nitrate response (Sato et al., 2017), nitrogen utilization (Xin et al., 2025), nodule formation regulation (Schauser et al., 1999), and stress response (Lian et al., 2022). In *Arabidopsis thaliana*, NLP7 can directly bind to nitrate, relieve autoinhibition by conformational changes, alter its subcellular localization, translocate into the nucleus, and bind to NRE elements in the promoters of target genes to participate in nitrate signal transduction (Liu et al., 2022). NLP7 also functions in the cold stress response. Cold stress triggers an increase in cytoplasmic Ca^2+^ concentration; CPK28 binds Ca^2+^ and subsequently phosphorylates *Arabidopsis* NLP7 protein, which promotes its rapid nuclear translocation, initiates transcriptional reprogramming of COR genes, and ultimately enhances plant cold tolerance (Ding et al., 2022). Another study indicated that *Arabidopsis* NLP8 can directly bind to the promoter of *CYP707A2*, promote abscisic acid degradation mediated by this gene, and thereby facilitate seed germination (Yan et al., 2016). In rice, OsNLP4 binds to the NRE element on the promoter of nitrite reductase to activate the expression of *OsNiR*, which improves nitrogen use efficiency, grain yield, and tiller number in rice (Yu et al., 2021). In *Lotus japonicus*, LjNLP3 has been identified as a key regulator of nodule development through spatiotemporal transcriptome profiling. LjNLP3 not only activates *LjLbs* to maintain the hypoxic microenvironment required for nitrogen fixation, but also forms dimers with LjNIN to repress the expression of *LjNF-Ys*, thereby terminating nodule cell differentiation and initiating nodule maturation (Ye et al., 2024).

With the continuous characterization of the functions of *NLP* gene family members, diverse biological functions have been identified within this family. To comprehensively clarify the functional characteristics of *NLP* family members, the *NLP* gene family has been identified in an increasing number of plant species at the whole-genome level, and their multiple functional roles have been systematically explored in several studies. Identification results show that both *Arabidopsis thaliana* and *Zea mays* contain nine *NLP* family members (Yan et al., 2016; Wang et al., 2018), *Populus euphratica* contains ten members (Long et al., 2026), *Triticum aestivum* contains 18 members (Mishra et al., 2025), *Brassica napus* contains 31 members (Liu et al., 2018), and *Setaria italica* contains seven members (Bai et al., 2024). Nevertheless, most of these studies have mainly focused on biological processes, such as nitrogen fixation, nitrate signal response, and temperature stress tolerance.

In contrast to previous studies, the present study innovatively explored the regulatory roles of *NLP* genes in ginsenoside biosynthesis in ginseng. By analyzing the gene structure, expression patterns, *cis*-acting regulatory elements, and phylogenetic relationships of *NLP* family members, combined with correlation analysis between phenotypic traits and gene expression levels, association analysis of SNP/InDel variations with phenotypic traits, and correlation analysis between the expression levels of key enzyme genes and *PgNLP* genes, two core genes regulating ginsenoside biosynthesis were identified. Furthermore, the expression patterns of these two genes in ginseng adventitious roots under MeJA treatment were investigated, and their correlation with ginsenoside content was analyzed. Among them, *PgNLP07* was identified as a positive regulator, while *PgNLP02–01* was identified as a negative regulator of ginsenoside biosynthesis. This study provides valuable genetic resources for comprehensively elucidating the functions of the *NLP* gene family in ginseng and offers a novel research direction for future functional studies of *NLP* genes in ginseng.

## 2 Materials and methods

### 2.1 Identification of the *PgNLP* gene family members

In this study, 348 protein sequences of the NLP family from 15 species were downloaded from the Plant Transcription Factor Database (https://planttfdb.gao-lab.org/). Gene set I was obtained by performing BLAST (version 2.1.7) searches against transcriptome datasets (NCBI, https://www.ncbi.nlm.nih.gov/) (BioProject accession:PRJNA302556) with an E-value cutoff of ≤1×10^-6^. The PF02042 and PF00564 files were downloaded from the InterPro website (https://www.ebi.ac.uk/interpro/entry/pfam/#table). The Hmmersearch module embedded in HMMER software (v3.3.2) was employed to screen the protein database of *Panax ginseng* (CNCB, https://www.cncb.ac.cn/) (BioProject accession: PRJCA022032), and an E-value of ≤ 0.01 was defined as the cutoff criterion for significant domain hits. The retrieved protein sequences were treated as query sequences for subsequent alignment against transcriptome datasets, and Gene Set II and Gene Set III were thereby acquired. The obtained protein sequences were used as queries to align against the transcriptome, and Gene Groups II and III were acquired. The common genes between Groups II and III were extracted and combined with Gene Group I. The remaining genes were considered as candidate genes for identification.

A Perl script was used to retrieve the nucleic acid sequences of the candidate genes, which were then submitted to NCBI CD-search (https://www.ncbi.nlm.nih.gov/Structure/cdd/wrpsb.cgi). Sequences containing both complete RWP-RK and PB1 domains were retained. TBtools (v2.468) was applied to extract full-length open reading frames (ORFs) from the retained sequences. The ORFs were submitted to SMART (https://smart.embl.de/) to re-verify the types and integrity of their conserved domains. The identified genes were renamed in the format *PgNLP*, followed by a numerical serial number.

### 2.2 Structural analysis of *PgNLP* genes

The amino acid sequences encoded by the ORFs of *PgNLP* genes were submitted to MEGAX64. A phylogenetic tree was constructed using the maximum likelihood (ML) method with the JJT+G model, and the bootstrap value was set to 2000 to analyze the subtypes of the family members. The amino acid sequences were submitted to MEME (https://meme-suite.org/meme/) to identify 10 conserved motifs and explore their composition and distribution of conserved motifs. The amino acid sequences were uploaded to Batch CD-search (https://www.ncbi.nlm.nih.gov/Structure/wrpsb-out/wrpsb.cgi) to obtain conserved domain information for *PgNLP* family members. Finally, TBtools was used to visualize the phylogenetic relationships, motif distribution, and conserved domain composition of the gene family members.

### 2.3 Chromosome localization and collinearity analysis of *PgNLP* genes

The sequences of *PgNLP* family members were blasted against the telomere-to-telomere (T2T) reference genome of *Panax ginseng* (CNCB, https://www.cncb.ac.cn/) (BioProject accession: PRJCA022032). The parameters were set as identity higher than 95% and alignment length longer than 1000 bp, and the chromosomal localization information of *PgNLP* genes were obtained. The online tool MG2C v2.0 (http://mg2c.iask.in/mg2c_v2.0/) was used to visualize chromosomal distribution. Meanwhile, with a threshold of identity ≥ 95% and alignment length ≥ 500 bp, the differential chromosomal localization information of homologous genes was screened and summarized. R software (v4.6.0) was used for collinearity visualization, and green connecting lines were used to represent similar gene fragments distributed at different chromosomal positions.

### 2.4 Phylogenetic analysis of *PgNLP* gene family

The ORF sequences of *PgNLP* gene family members were subjected to phylogenetic analysis, together with 21 genes from seven exogenous species (including one model plant, three monocotyledons, and three dicotyledons) (**Supplementary Table 2**). Amino acid sequences were aligned using MEGA software. Subsequently, a phylogenetic tree was constructed using the maximum likelihood (ML) method with the JJT+G+I model, and the bootstrap value was set to 2000. Finally, the phylogenetic tree was visualized using the online tool ChiPlot (https://www.chiplot.online/) (Xie et al., 2023).

### 2.5 Expression pattern analysis of *PgNLP* genes

The expression levels of *PgNLP* family members were extracted using R (v4.6.0) from three transcriptome datasets, including the main root expression profiles of *Panax ginseng* at four different growth years, 14 tissue parts of five-year-old ginseng, and the main root expression data of 42 landrace ginseng accessions (PRJNA302556). Gene expression values were normalized and clustered using TBtools, and corresponding heatmaps were constructed.

### 2.6 Cis-acting elements analysis of *PgNLP* genes

Based on the chromosomal localization results, 2000 bp promoter sequences upstream of each gene were extracted. These nucleic acid sequences were submitted to PlantCARE (https://bioinformatics.psb.ugent.be/webtools/plantcare/html/) to analyze the distribution of cis-acting elements. The classification and quantity of cis-acting elements were visualized using the ggplot2 package, and their distribution patterns were displayed using TBtools software.

### 2.7 Association analysis of *PgNLP* genes SNP/InDel and ginsenoside content

The SNP/InDel variation information of *PgNLP* family members was retrieved from a laboratory-established SNP/InDel database containing 346 ginseng accessions(NCBI, https://www.ncbi.nlm.nih.gov/) (BioProject accession: PRJNA302556) (Liu et al., 2023). The homozygous reference allele genotype was defined as 0, the heterozygous genotype as 1, and the homozygous variant allele genotype as 2. Association analysis was performed between the genotypes of the variant loci and the contents of the nine ginsenosides in the tested samples. For variant loci with only two genotype types and each genotype containing more than three samples, the car package in R software was used for the homogeneity of variance test. If the variance was homogeneous, the independent sample *t*-test was adopted to detect the mean difference in ginsenoside content between the two groups; otherwise, the Welch-corrected *t*-test was applied. For loci with three genotype types that met the sample size requirement, one-way analysis of variance (ANOVA) with Type III sum of squares was conducted, and the Tukey method was used for *post hoc* multiple comparisons. A *p*-value ≤ 0.05 was regarded as the threshold for a significant effect of gene SNP/InDel on the corresponding ginsenoside accumulation. Genes harboring significantly associated variant loci were defined as the candidate gene cluster A.

### 2.8 Correlation analysis between *PgNLP* genes expression and ginsenoside content

Spearman correlation analysis was performed using SPSS (v26.0) to evaluate the relationship between the expression levels of *PgNLP* genes in the main roots of the 42 ginseng landraces and the contents of nine ginsenosides. A correlation with *p* ≤ 0.05 was considered statistically significant, and genes with significant correlations were inferred to participate in the regulatory process of ginsenoside biosynthesis. The obtained *p*-values and correlation coefficient (ρ) values were visualized by constructing a correlation heatmap. Genes with significant correlations were defined as the related gene cluster B.

### 2.9 Co-expression network analysis of *PgNLP* genes and key enzyme genes involved in ginsenoside biosynthesis

Spearman correlation analysis was performed based on the expression levels of *PgNLP* genes and 17 key enzyme genes involved in the ginsenoside biosynthesis pathway, which had been previously cloned and functionally validated in *Panax ginseng*. The resulting data matrix was imported into BioLayout Express ^3D^ for network visualization. Genes are represented as nodes, and correlation relationships are indicated by edges. Co-expression networks were constructed using significance thresholds of *p* = 0.05, *p* = 0.01, and *p* = 0.001. *PgNLP* genes that maintained close interaction networks with key enzyme genes at a threshold of *p* = 0.001 were defined as related gene cluster C.

### 2.10 Quantitative real-time PCR analysis of *PgNLP* genes and key enzyme genes, and determination of ginsenoside contents in ginseng adventitious roots under MeJA treatment

Adventitious roots of Panax ginseng proliferated in liquid Gamborg’s B5 medium were treated with 200 μM methyl jasmonate (MeJA). Sampling was performed at the untreated control time point and at 6 h, 12 h, 24 h, 36 h, 48 h, 60 h, 72 h, 84 h and 96 h post-treatment. Three biological replicates were set for each sampling time point; specifically, three randomly selected bottles of proliferated adventitious roots were harvested as independent biological samples at each time point, which were subsequently used for total RNA extraction and ginsenoside content determination. Total RNA was isolated from ginseng adventitious roots using the Trizol method and reverse-transcribed into complementary DNA (cDNA). Quantitative detection of the finally screened PgNLP genes and key enzyme genes was carried out on the ABI 7500 Real-Time PCR System. Three technical replicates were conducted for each biological replicate. The CYP gene was adopted as the internal reference gene (Li et al., 2019), and the 2^−ΔΔCt^ method was applied to calculate the relative expression levels of target genes. Using the untreated group as the control, one-way analysis of variance was performed on the relative expression levels of *PgNLP* genes at each time point after treatment with MeJA. The remaining ginseng adventitious roots were dried at low temperature, ground into powder, and subjected to water extraction for ginsenosides, followed by purification using an ODS column. The extracted ginsenoside sample was separated into individual ginsenosides using a Waters e2695 separation module with a Waters C18 column. The mobile phase consisted of acetonitrile (A) and Wahaha purified water (B). The separation time was 120 minutes, with gradient elution (**Supplementary Table 5**) (Li et al., 2019; Li et al., 2021; Liu et al., 2023). The injection volume was 10 µL, the column temperature was 30 °C, and the flow rate of the mobile phase was 1.0 mL/min. Three technical replicates were also performed for each biological replicate. A Waters e2498 UV detector was used to monitor the absorption peaks of ginsenosides at 203 nm, and the contents of ginsenosides Rb1, Rg2, Rh1, Rc, Rb2, Rb3, Rd, and Rg3 were calculated based on peak areas.

### 2.11 Correlation analysis of *PgNLP* genes and ginsenoside content under MeJA treatment

Based on the relative expression levels of *PgNLP* genes under MeJA induction and the ginsenoside content of the corresponding samples, Spearman correlation analysis was performed using SPSS (v23.0) software. The significance, correlation coefficients, and correlation relationships between genes and ginsenosides were visualized using OmicStudio (https://www.omicstudio.cn/tool) (Lyu et al., 2023).

### 2.12 Expression pattern analysis of *PgNLP* and key enzyme genes under MeJA treatment

Correlation and co-expression network analyses were conducted on the relative expression levels of *PgNLP* and key enzyme genes under MeJA treatment. We hypothesized that genes interacting directly in the ginsenoside biosynthesis pathway would exhibit significantly higher correlation coefficients than those with indirect interactions. Based on this assumption, a putative regulatory network of *PgNLP* genes involved in ginsenoside biosynthesis was established. The core *PgNLP* genes were defined as hub genes, and other genes were connected to these hub genes based on their correlation coefficients. The potential regulatory network was then plotted.

## 3 Results

### 3.1 Structural analysis of *PgNLP* genes

**Figure 1:**
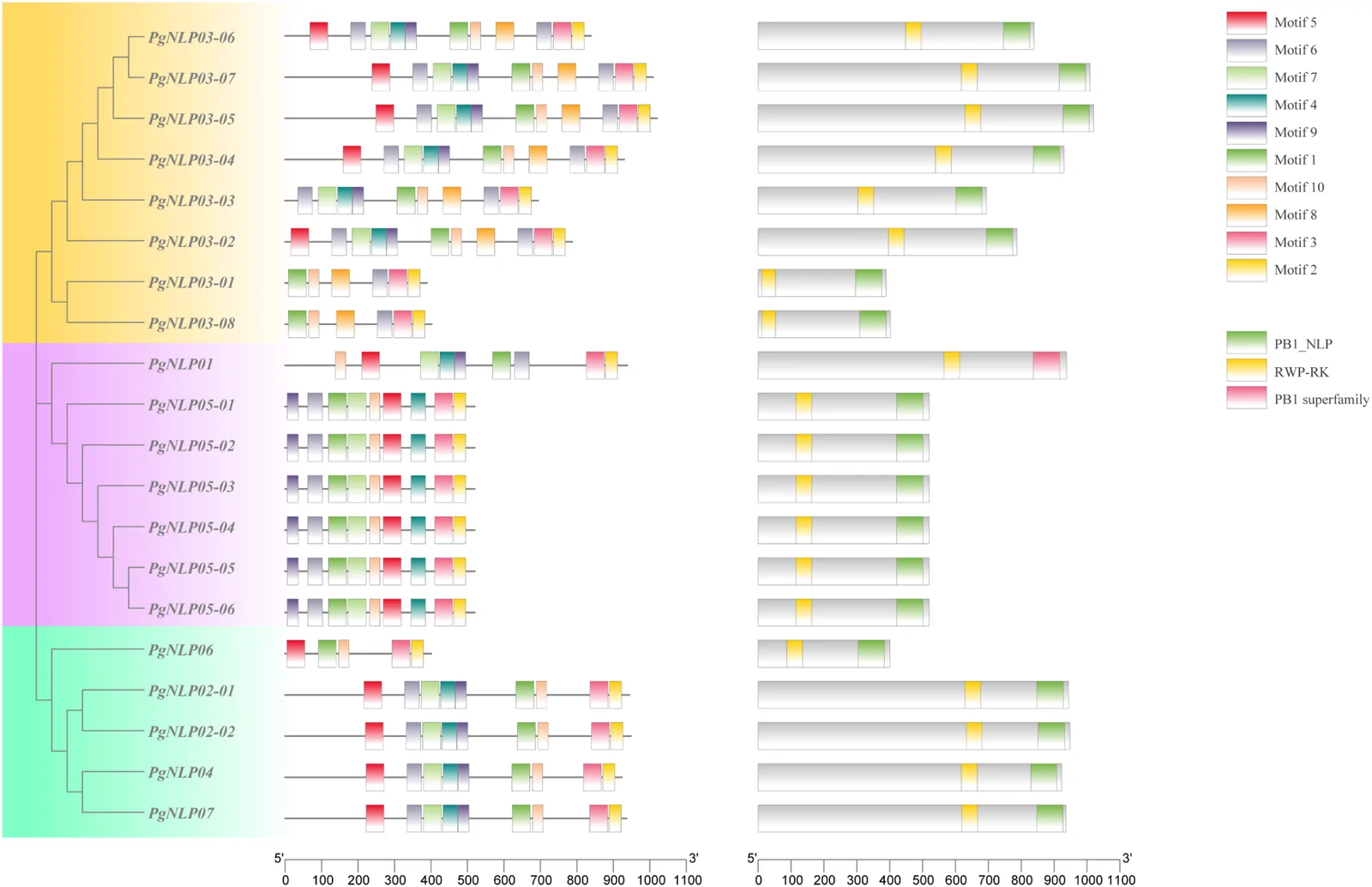
Phylogenetic analysis, motif distribution and conserved domain analysis of *PgNLP* gene family members. The phylogenetic tree, *PgNLP* members shaded in yellow belong to the NLP_A subtype, those shaded in purple represent NLP_B, and those shaded in green correspond to NLP_C. The phylogenetic tree was constructed using the Maximum-likelihood (ML) method with the JTT+G model.

A total of 20 members of the ginseng *NLP* gene family were identified through homologous comparison and protein conserved domain analysis. All 20 genes contained the RWP-RK domain together with either the PB1 or PB1 superfamily domain, exhibiting typical structural characteristics of NLP proteins (**Supplementary Table 1**). These *PgNLP* members were classified into three subtypes based on phylogenetic analysis. *PgNLP01* showed a close phylogenetic relationship with *PgNLP05*, whereas *PgNLP02* was closely related to *PgNLP06*. In contrast, *PgNLP03* clustered into an independent clade. Consistent regularities were also observed in the distribution patterns of the motifs. Motifs 2 and 3 were detected in all PB1 domains, whereas motif 1 was universally present in the RWP-RK domains. Genes belonging to the NLP_A subtype displayed a tandem arrangement of motif1-10-8-6-3-2. NLP_B subtype genes lacked this conserved arrangement and were characterized by the combination motif10-5-4-3-2. NLP_C subtype genes harbored the structural pattern motif5-1-10-3-2. Notably, motif 8 was uniquely distributed in the NLP_A subtype genes, which might account for the formation of their independent phylogenetic clade (**Figure 1**).

### 3.2 Chromosome localization and collinearity analysis of *PgNLP* genes

**Figure 2:**
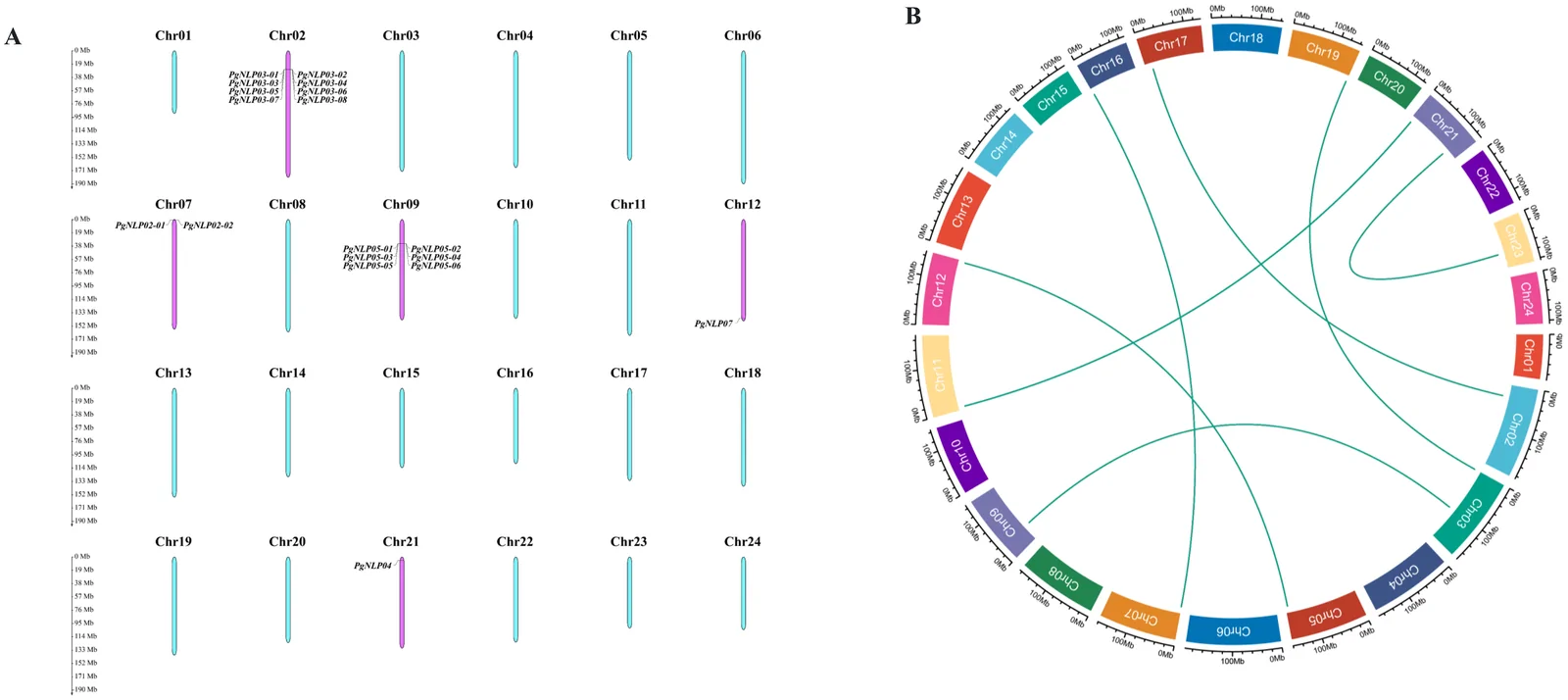
Chromosomal localization and distribution of *PgNLP* genes. **(A)** The scale on the left represents the physical position of chromosomes. Chromosomes mapped with *PgNLP* genes are filled in purple, while the remaining chromosomes are filled in green. **(B)** Intraspecific collinearity and duplication event analysis of the *PgNLP* gene family. The outer color blocks indicate chromosome number and physical position, and green curves link the chromosomal positions of collinear *PgNLP* gene pairs.

Comparative genomic analysis revealed that the 18 *PgNLP* members were unevenly distributed across five chromosomes of *Panax ginseng*. *PgNLP03* on chromosome 2 possessed eight transcripts, whereas *PgNLP05* on chromosome 9 possessed six transcripts. Meanwhile, *PgNLP02*, located in the subtelomeric region of chromosome 7, contained two transcripts. These phenomena were most likely caused by alternative splicing during transcription (**Figure 2A**). In addition, *PgNLP01* and *PgNLP06* failed to map to any chromosome in the reference genome. This might be attributed to the allotetraploid nature of ginseng, which harbors abundant presence–absence variations. Differences in the sequencing materials used for the reference genome and transcriptome also led to the detection of distinct gene sequences.

The divergence pattern of the *NLP* gene family was characterized by statistically counting the differential chromosomal loci of each gene (**Figure 2B**). The results indicated that tandem duplication events were rarely detected in the ginseng *NLP* gene family. Chromosomal localization showed that several family members were distributed near both ends of the chromosomes. Collinearity analysis further demonstrated that *PgNLP* genes located on chr07/chr16, chr05/chr12, chr11/chr21, and chr03/chr09 were positioned in telomere-proximal chromosomal regions. In contrast, *PgNLP* genes on chr21/chr23 and chr03/chr09 were distributed in euchromatin-enriched gene clusters in the middle regions of chromosomes, which was consistent with the chromosomal distribution pattern of the *PgNLP* gene family. This evolutionary pattern probably resulted from interchromosomal crossover and recombination among non-homologous chromosomes during gene family evolution, which drove the expansion of the *PgNLP* gene family.

### 3.3 Phylogenetic analysis of *PgNLP* genes

**Figure 3:**
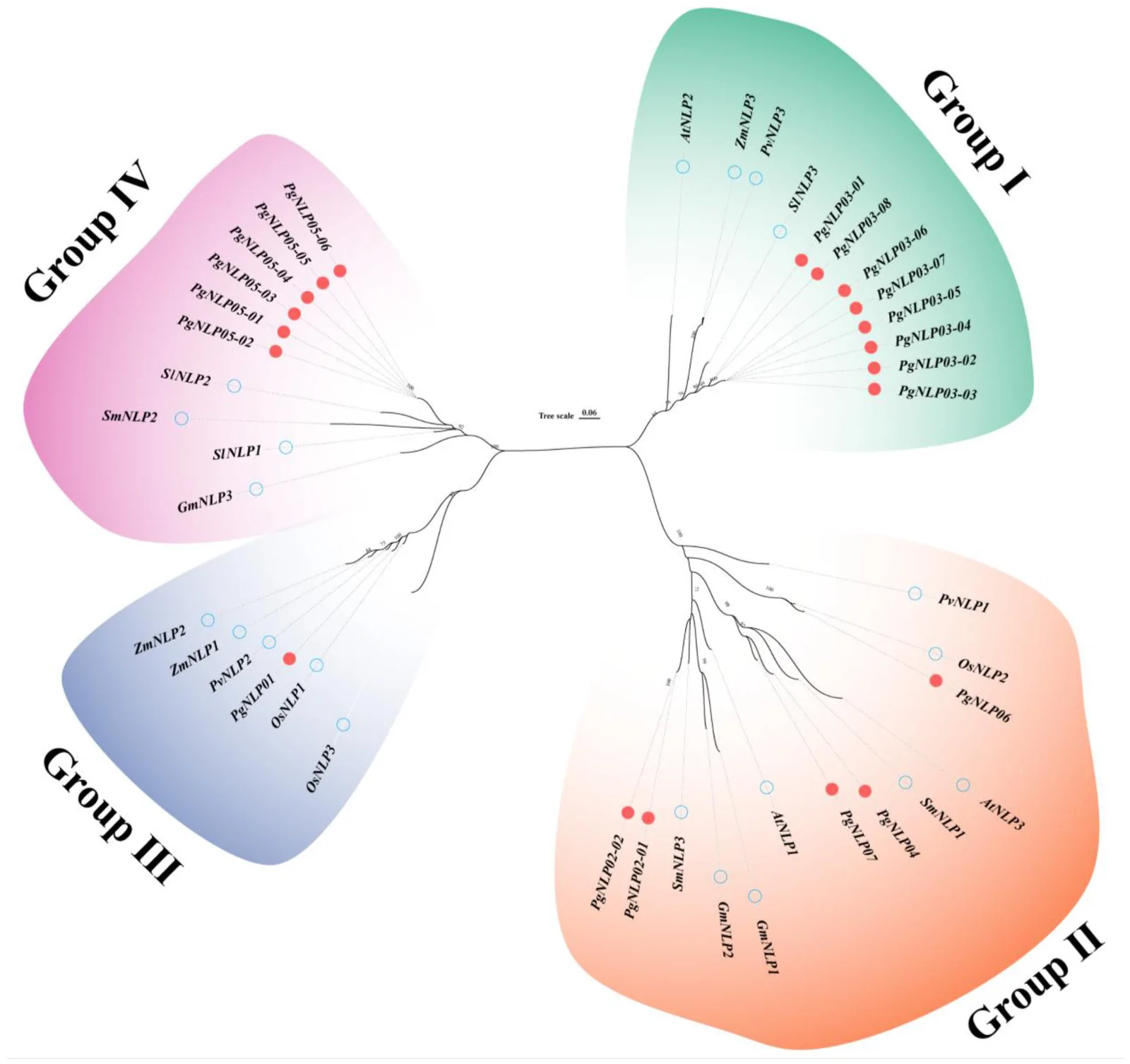
Phylogenetic tree analysis of the NLP gene family among *Panax ginseng*, *Arabidopsis thaliana*, *Glycine max*, *Solanum lycopersicum*, *Salvia miltiorrhiza*, *Zea mays*, *Oryza sativa*, and *Panicum virgatum*. Red solid circles mark NLP members of P ginseng, and blue hollow circles represent NLP members from other exogenous species. All members were classified into distinct subclades according to evolutionary distances, which clarified the evolutionary status, subfamily classification of PgNLP genes, and their phylogenetic relationships with homologous genes from other plant species.

Phylogenetic analysis classified *PgNLP* genes into four clades, and the clustering pattern was largely consistent with the phylogenetic tree derived from gene structure analysis. The only difference was that *PgNLP01* independently clustered with monocotyledonous species to form Group III, whereas it still maintained a close phylogenetic relationship with *PgNLP05* in Group IV (**Figure 3**). This finding indicates that the unique structure of *PgNLP01* is not specific to *Panax ginseng* but represents a subtype shared by monocotyledons. This gene may be a retained single-copy conserved gene during the extensive lineage-specific gene loss event in eudicots (De Smet et al., 2013). Multiple *PgNLP03* and *PgNLP05* genes were distributed in separate clades, suggesting that these two genes possess substantial differences in sequence structure and function. *PgNLP02*, *PgNLP04*, and *PgNLP06* clustered into the same group, implying their similar roles in functional differentiation. This result also provided molecular evidence that *PgNLP02–01* and *PgNLP07* jointly responded to methyl jasmonate induction and regulated ginsenoside biosynthesis.

### 3.4 Expression pattern analysis of *PgNLP* genes

**Figure 4:**
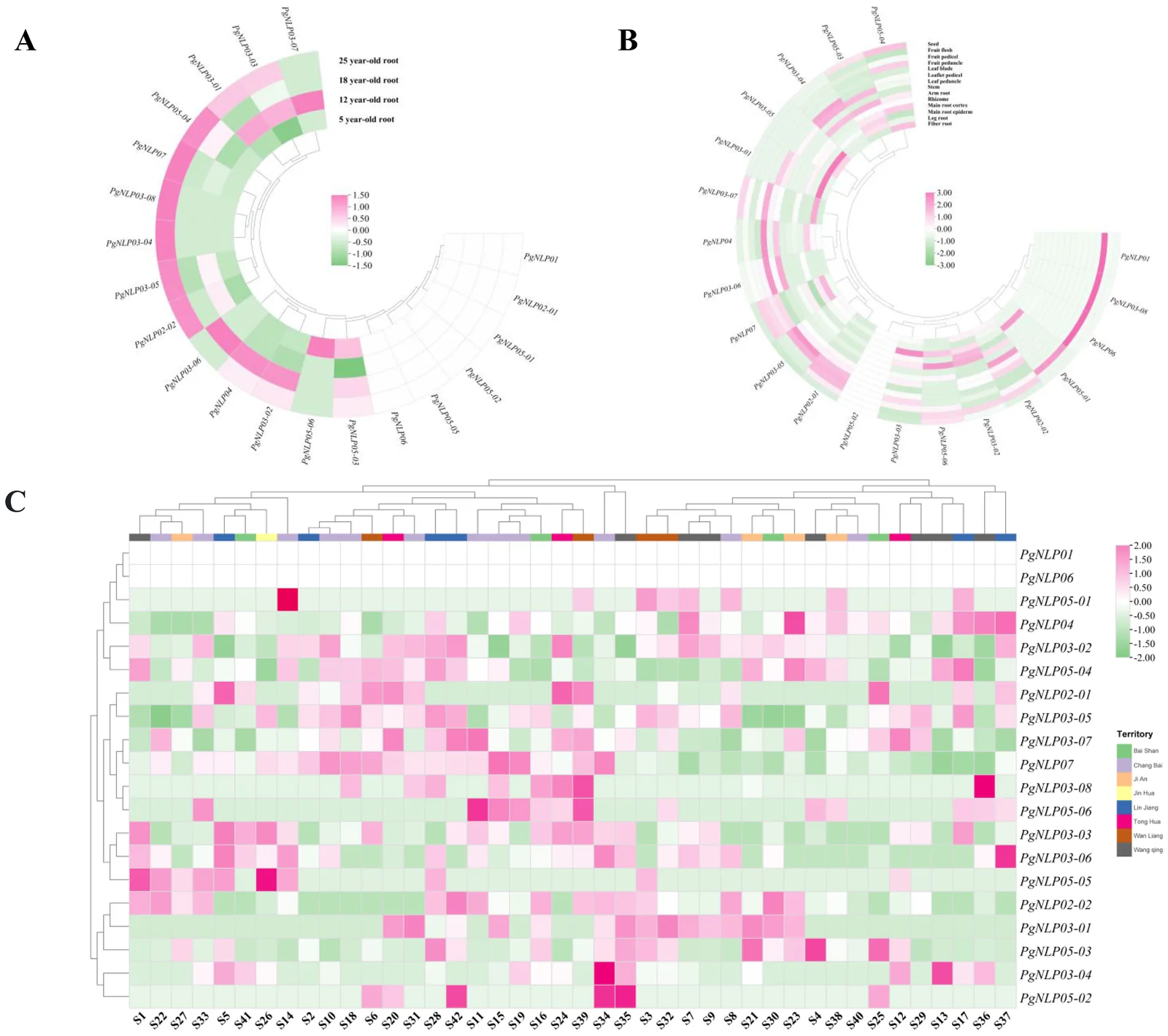
Expression characteristics and spatiotemporal expression patterns analysis of *PgNLP* genes in ginseng. **(A)** Expression patterns of *PgNLP* genes in roots of ginseng at four different growth years. **(B)** Expression patterns of *PgNLP* genes in 14 tissues of four-year-old ginseng. **(C)** Expression patterns of *PgNLP* genes in roots of 42 ginseng landraces. All expression levels were normalized in the analysis, and clustering was performed using the Markov clustering algorithm.

*PgNLP01* and *PgNLP06* were not expressed in the main roots of either the 42 ginseng landraces or ginseng roots at four different growth years (**Figure 4A**). In contrast, they exhibited specific expression in fruit pedicels, indicating that these two genes did not function in roots. It was speculated that they participated in nitrogen regulation in fruit pedicels, thereby affecting the development of fruits and seeds (**Supplementary Table 3**). By comparing the average expression levels of *PgNLP* genes in aboveground and underground tissues across 14 tissue sites, we found that the expression of *PgNLP02–01* and *PgNLP03–06* was significantly higher in the six underground tissues than in the eight aboveground tissues (**Figure 4B**). This result suggests that these two genes play essential roles in root development and underground nitrogen fixation in ginseng. In the main roots of 42 ginseng landraces, eight family members (*PgNLP03-02*, *PgNLP03-03*, *PgNLP03-05*, *PgNLP03-07*, *PgNLP04*, *PgNLP07*, and *PgNLP05-04*) showed TPM values greater than 1. Interestingly, genes expressed in more than 75% of the landraces all belonged to the *PgNLP03*, *PgNLP04*, and *PgNLP05* lineages (**Figure 4C**). Their constitutive and stable expression in main roots implied that they acted as housekeeping genes and participated in the regular growth and development of ginseng roots. Nevertheless, both *PgNLP02–01* and *PgNLP02–02* exhibited relatively low expression levels. This low expression did not indicate that these genes were functionally inactive; instead, they were more likely to be inducible genes involved in stress adaptation and signal-response processes.

### 3.5 Cis-acting elements analysis of *PgNLP* genes

**Figure 5:**
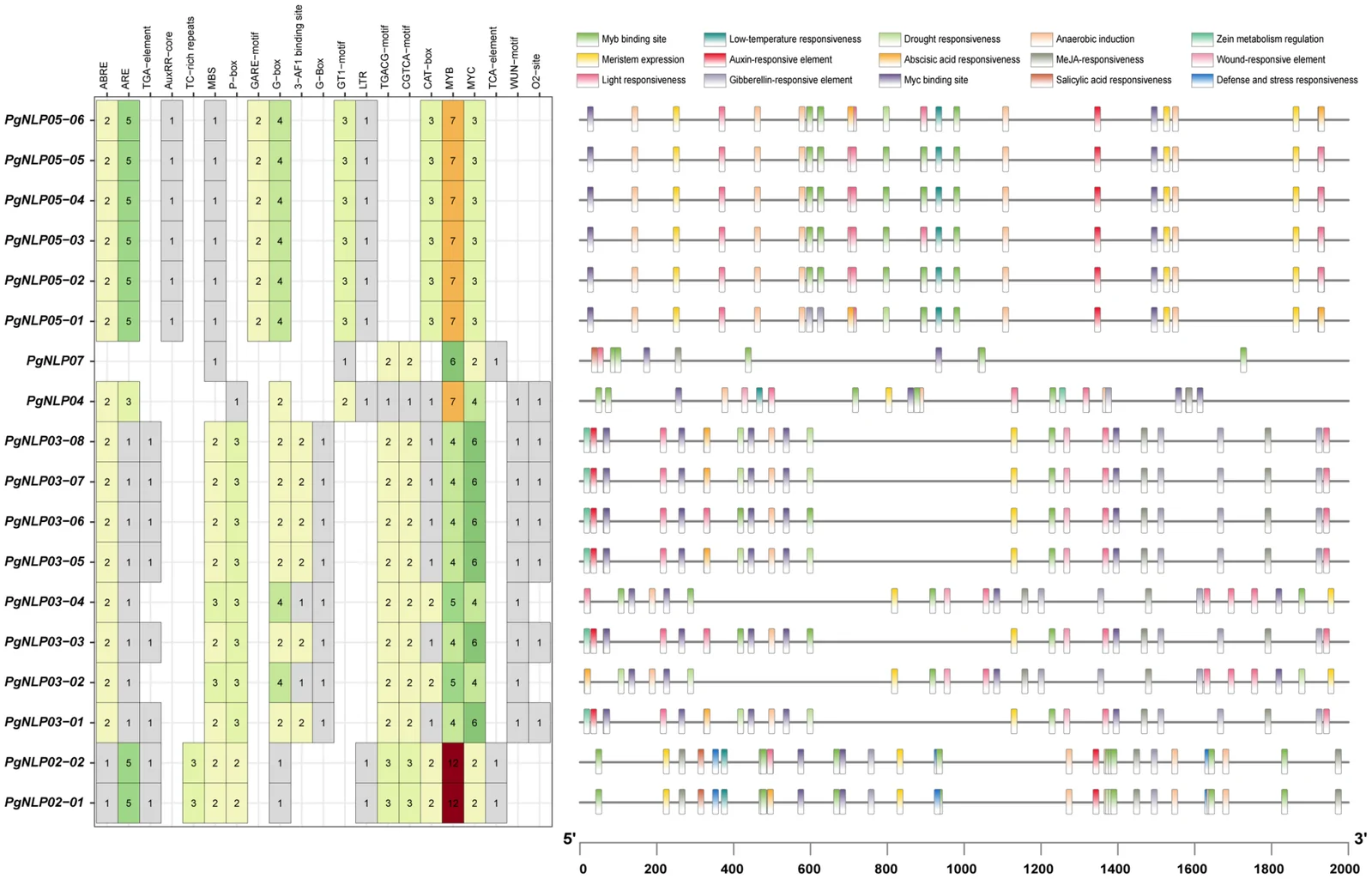
Statistics and distribution analysis of cis-acting elements in the promoters of *PgNLP* genes. The left panel shows the statistics of the types and numbers of cis-acting elements, and the right panel visualizes the physical distribution of cis-acting elements along the promoter regions.

Cis-acting element analysis showed that all *PgNLP* family members harbored cis-acting elements associated with meristem expression, providing novel insights into the potential roles of *NLP* genes in ginseng root growth and development. Anaerobic induction elements were widely distributed in the promoter regions of *PgNLP* genes. All members, except *PgNLP07*, contained 1–5 copies of this element, suggesting that *PgNLP* genes may play vital roles in defending against anaerobic stress under waterlogging conditions. Notably, the upstream promoters of *PgNLP02*, *PgNLP03*, and *PgNLP04* possessed methyl jasmonate (MeJA)-responsive elements, with copy numbers ranging from 2 to 6 (**Figure 5**). Previous studies have demonstrated that exogenous MeJA induction effectively increases ginsenoside accumulation in ginseng plants, adventitious roots, and callus tissues (Kim et al., 2004). This evidence further indicates that *PgNLP* family members may be induced by MeJA and subsequently regulate ginsenoside biosynthesis.

### 3.6 Association analysis of *PgNLP* gene SNP/InDel and ginsenoside content

**Figure 6:**
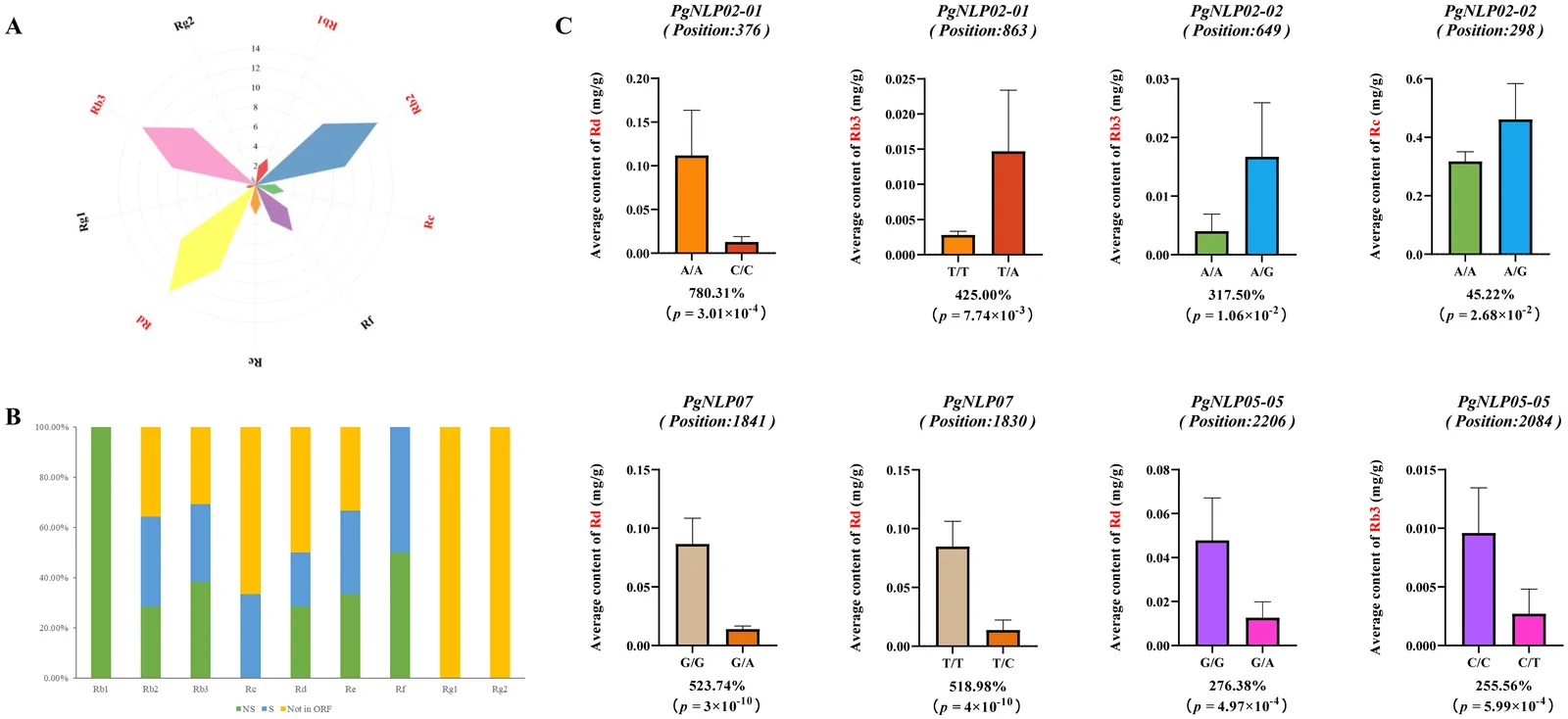
The regulatory effects analysis of SNP/InDel in the *PgNLP* genes on ginsenosides. **(A)** Significantly affect the distribution of the number of loci for each ginsenoside monomer. **(B)** Significantly affect the effect and proportion of each ginsenoside locus. **(C)** The biological effects of significantly influential SNPs/InDels on the each ginsenoside monomer content.

A total of 382 mutation sites of *PgNLP* genes were retrieved from the SNP/InDel database, containing 346 *Panax ginseng* accessions. Association analysis between SNP/InDel variations of family members and the contents of nine ginsenosides identified 26 significant loci distributed in four genes, *PgNLP02-01*, *PgNLP02-02*, *PgNLP07*, and *PgNLP05-05*, which showed significant differences in ginsenoside content among different genotype groups (**Figure 6C**, **Supplementary Table 4**). Among these loci, two SNPs at 1830 and 1841 bp of *PgNLP07* exerted significant effects on the contents of five ginsenosides (Rb2, Rb3, Rd, Re, and Rf). Statistical analysis of the significant SNP/InDel numbers associated with each ginsenoside subtype identified 26 significant loci related to protopanaxadiol-type ginsenosides (Rb1, Rb2, Rb3, Rc, and Rd), whereas only nine significant loci were detected for protopanaxatriol-type ginsenosides (**Figure 6A**). This result indicates that *PgNLP* genes are more likely to participate in the regulation of protopanaxadiol-type ginsenoside biosynthesis. Among the 26 SNP/Indel loci that significantly affected the nine ginsenosides, 36.2% were located outside the open reading frame (ORF) and did not alter the amino acid sequences of the encoded proteins. These non-coding variations may affect phenotypic variation by regulating mRNA stability. In addition, 31% of the loci caused non-synonymous mutations, which could change the protein structure and hydrophobicity and further influence protein function. The remaining 32.8% of loci belonged to synonymous mutations (**Figure 6B**). According to recent studies, synonymous mutations can also regulate phenotypic traits by modulating m6A modification and mRNA conformational structure (Xin et al., 2025).

### 3.7 Correlation analysis between *PgNLP* genes expression and ginsenoside content

**Figure 7:**
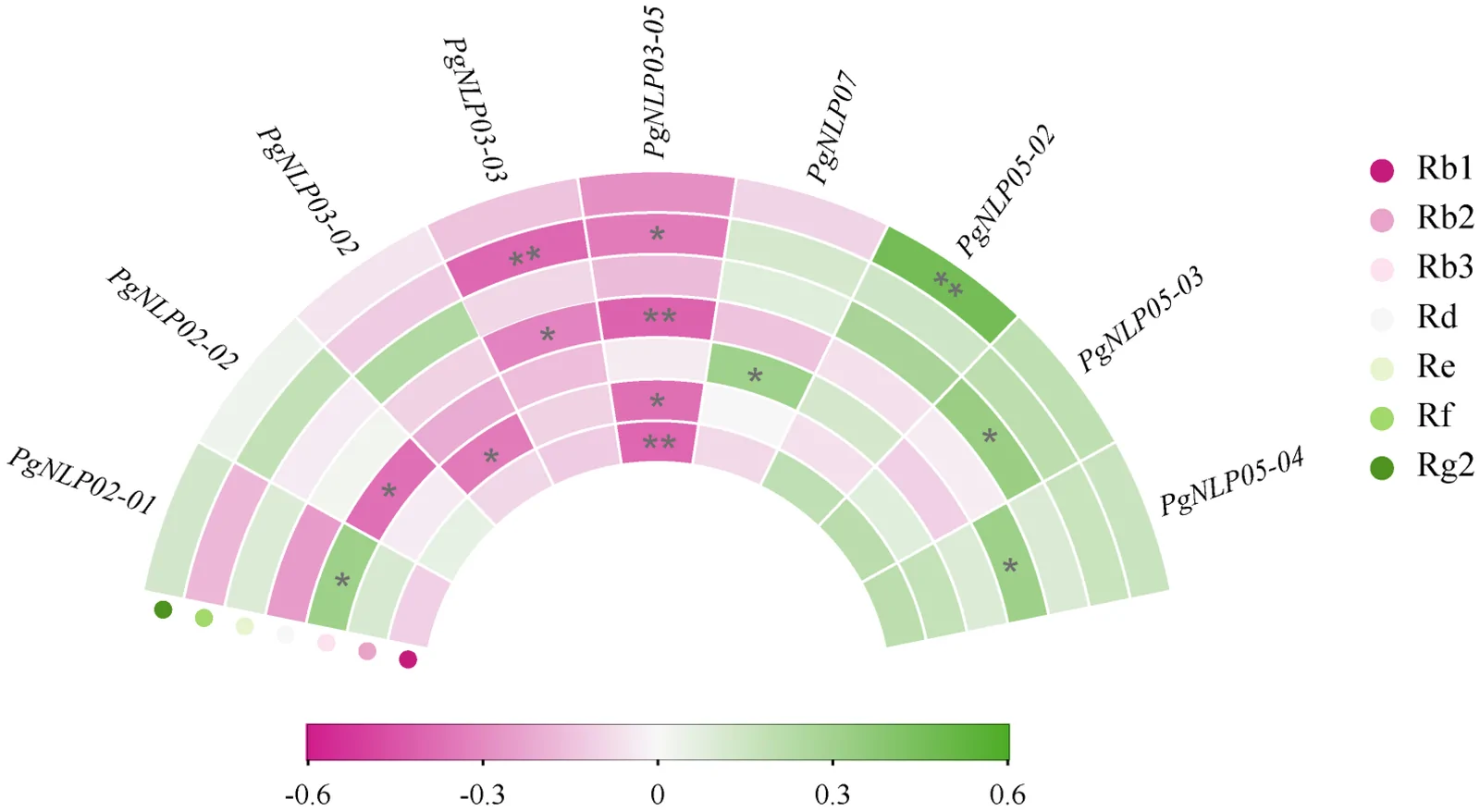
Correlation analysis between *PgNLP* genes expression and the contents of nine ginsenosides in 42 ginseng landraces. Only genes and ginsenosides with significant correlations are presented in the figure. Asterisk * indicates significant correlation at p ≤ 0.05, double asterisks ** indicate extremely significant correlation at p ≤ 0.01.

Correlation analysis identified nine *PgNLP* genes (*PgNLP02-01*, *PgNLP02-02*, *PgNLP03-02*, *PgNLP03-03*, *PgNLP03-05*, *PgNLP04*, *PgNLP05-02*, *PgNLP05-03*, and *PgNLP05-04*) whose expression levels were significantly correlated with the nine ginsenoside contents. Among them, the expression of *PgNLP05–03* showed a significant negative correlation with the four ginsenosides. Statistical analysis revealed that all significantly correlated transcripts of *PgNLP03* exhibited negative correlations with ginsenoside content, whereas those associated with PgNLP05 exhibited positive correlations (**Figure 7**). Combined with the previous expression pattern analysis, *PgNLP03* and *PgNLP05* were inferred to be constitutive genes involved in regulating ginsenoside biosynthesis during normal growth and development of ginseng plants. Both transcripts *PgNLP02–01* and *PgNLP02–02* genes were significantly correlated with Rb3. Notably, the two transcripts derived from the same gene displayed correlation patterns in opposite directions, indicating that these two transcripts possessed distinct biological functions and triggered different biological effects (Ning et al., 2025).

### 3.8 Co-expression network analysis of *PgNLP* genes and key enzyme genes involved in ginsenoside biosynthesis

**Figure 8:**
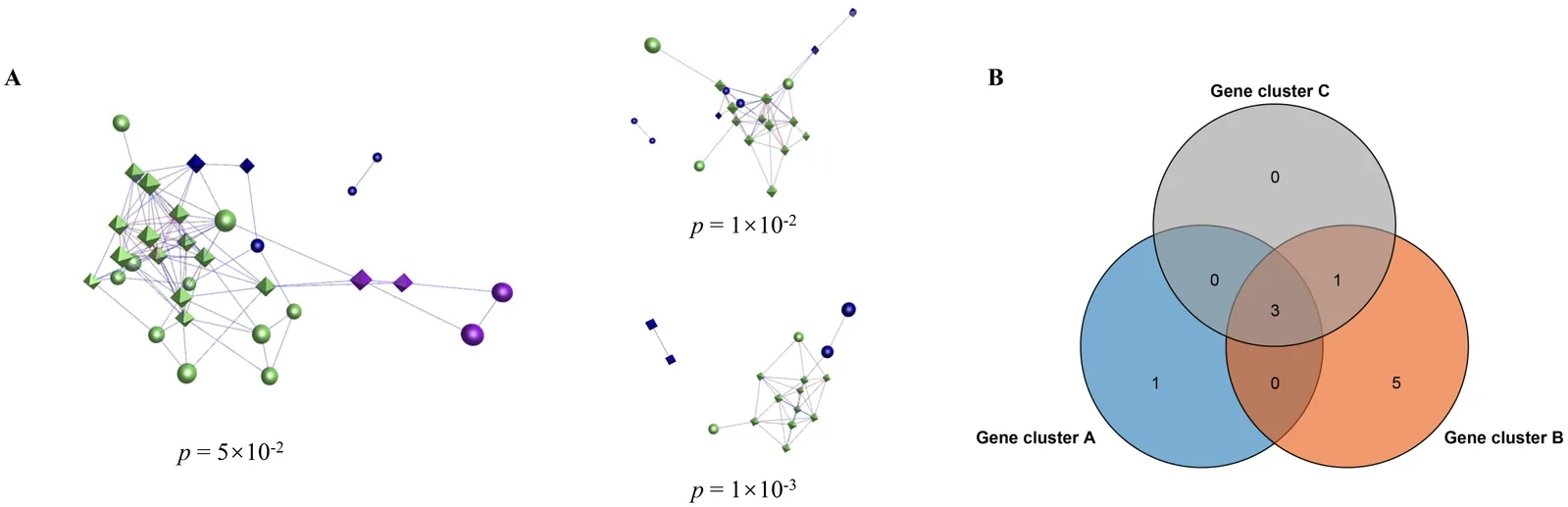
Co-expression network analysis of *PgNLP* genes with ginsenoside biosynthetic enzyme genes. **(A)** Co-expression network analysis of expression levels between *PgNLP* genes and 17 key enzyme genes in 42 ginseng landraces. Regular hexahedrons represent key enzyme genes, and spheres represent *PgNLP* genes. The green modules indicate genes clustered into the same cluster with consistent expression trends. **(B)** Sets of genes significantly associated with ginsenoside biosynthesis. Candidate genes were identified by intersecting gene groups obtained from three independent screening approaches.

Correlation analysis of the expression levels of *PgNLP* genes and key enzyme genes involved in the ginsenoside biosynthesis pathway showed that 15 *PgNLP* genes formed a close interaction network with 17 key enzyme genes at a threshold of *p* = 0.05, which were divided into two clusters. At *p* = 0.01, four *PgNLP05* transcripts that previously participated in the close interaction network were no longer retained in the network. At this threshold, seven *PgNLP* genes constructed a co-expression network with 15 key enzyme-encoding genes. When the *p*-value was further narrowed to 0.001, only four genes (*PgNLP02-01*, *PgNLP02-02*, *PgNLP03-05*, and *PgNLP07*) formed a network with 12 key enzyme genes. After Markov clustering algorithm analysis, *PgNLP03–05* and *PgNLP07* were clustered into the same group as ten key enzyme genes, indicating that these 12 genes shared similar expression patterns (**Figure 8A**).

Subsequently, Gene cluster A, obtained from the association analysis between *PgNLPs* SNPs/InDels and ginsenoside contents, gene cluster B, with significant correlations to the nine ginsenosides, and gene cluster C, exhibiting significant co-expression trends with key enzyme genes, were intersected. Three core genes were identified from the intersection of the three sets (**Figure 8B**). These three genes showed significant associations with ginsenoside biosynthesis at the genetic variation, gene expression, and co-expression levels with key enzymes. More than four MeJA-responsive cis-acting elements were detected in the promoter region of each gene. Finally, the expression profiles of *PgNLP02-01*, *PgNLP02-02*, and *PgNLP07* were determined in ginseng adventitious roots following MeJA treatment, and their correlations with ginsenoside accumulation were analyzed to infer their potential regulatory mechanisms.

### 3.9 The qRT-PCR of *PgNLP* genes and key enzyme genes in ginseng adventitious roots under MeJA treatment

**Figure 9:**
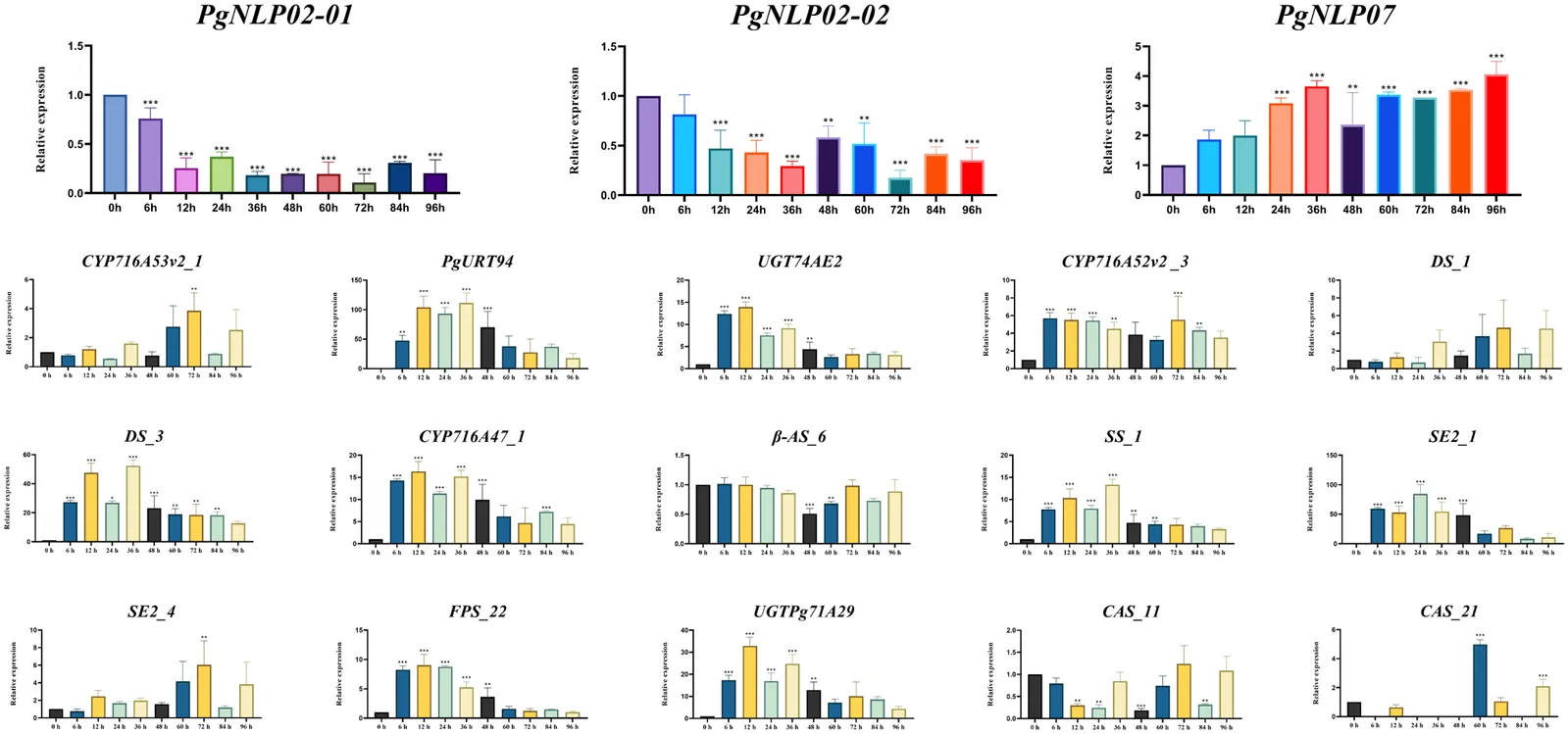
Expression patterns of three candidate genes and fifteen key enzyme genes in *Panax ginseng* adventitious roots under methyl jasmonate (MeJA) treatment. Intergroup differences were analyzed via one-way ANOVA, followed by the LSD post-hoc multiple comparison test. Asterisks denote significant differences: *p≤ 0.05, **p ≤ 0.01, ***p ≤ 0.001.

Quantitative fluorescence analysis was performed to detect the expression patterns of *PgNLP02-01*, *PgNLP02-02*, *PgNLP07*, and 15 key enzyme genes in ginseng adventitious roots at different time points after MeJA treatment. The results showed that the expression levels of the three *PgNLP* genes changed significantly after methyl jasmonate treatment. The expression of *PgNLP02* was continuously downregulated with prolonged treatment time, whereas *PgNLP07* was markedly upregulated during this process. These expression alterations were consistent with the prediction of cis-acting elements, in which MeJA-responsive elements were identified in the upstream promoter regions of these genes. Among the 15 key enzyme genes, 14 exhibited significant changes in expression after MeJA treatment. Although *DS_1* expression did not show a significant response to MeJA, it was included in the subsequent regulatory network inference because it functions as an essential enzyme in the ginsenoside biosynthesis pathway. Of these key enzymes, 12 were significantly upregulated and 2 were downregulated after MeJA treatment (**Figure 9**). Two genes, *β-AS_6* and *CAS_11*, catalyze the conversion of 2,3-oxidosqualene into β-amyrin and sterol compounds, respectively. Under MeJA induction, the accumulation of triterpenoids increased significantly, whereas the sterol biosynthesis pathway was suppressed, which could be attributed to the downregulated expression of key enzymes involved in sterol biosynthesis.

### 3.10 Correlation analysis of *PgNLP* genes and ginsenoside content under MeJA treatment

**Figure 10:**
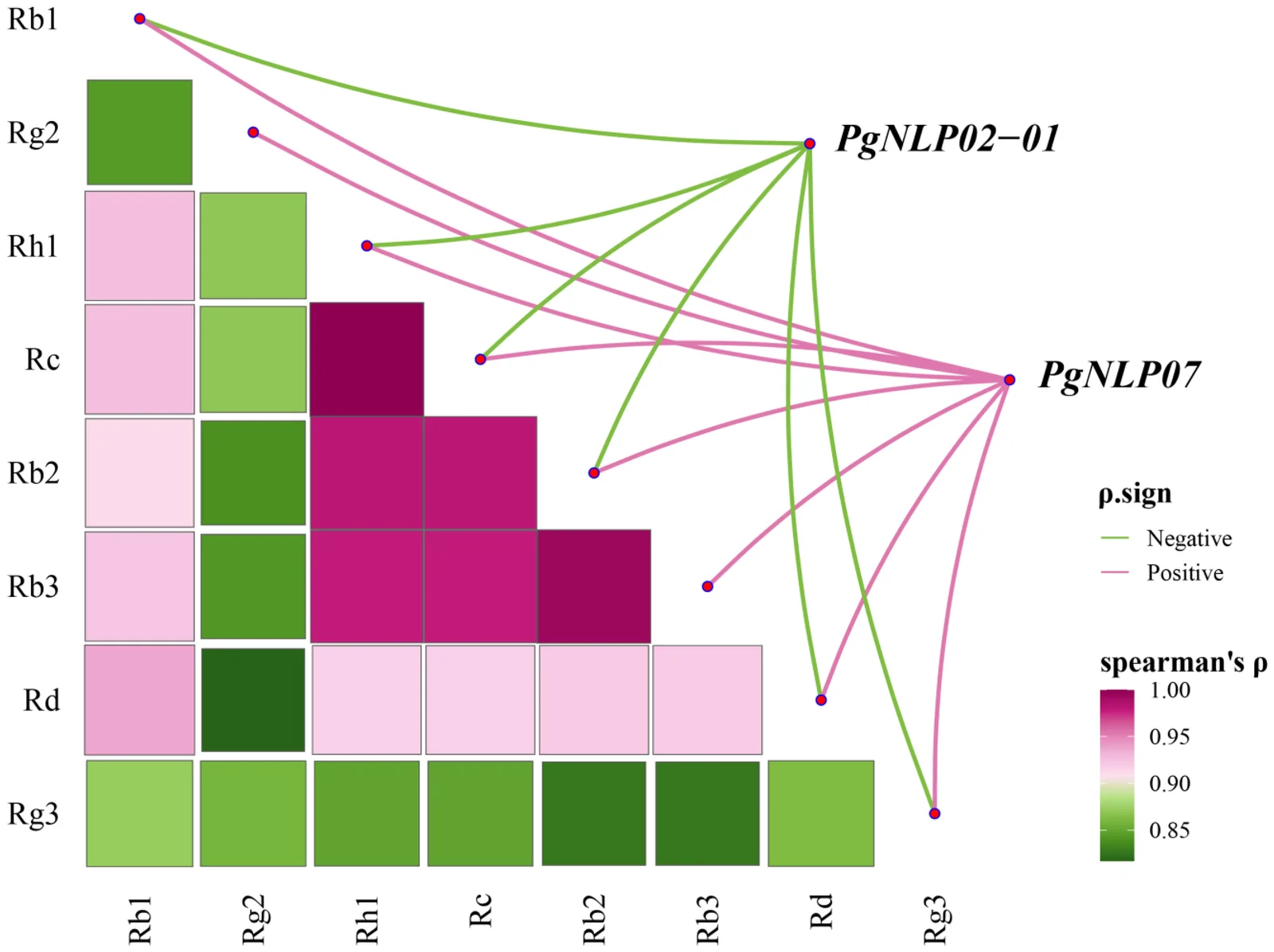
Correlation analysis between *PgNLP* genes expression and the contents of eight monomeric ginsenosides under MeJA induction. The color of the connecting lines between genes and ginsenosides indicates positive or negative correlation. All displayed connecting lines represent significant correlations between gene expression and ginsenoside content at p ≤0.05.

Correlation analysis revealed that although *PgNLP02–02* responded to MeJA treatment, its expression level was not correlated with the contents of the eight monomeric ginsenosides. Under MeJA induction, *PgNLP02–01* exhibited significant negative correlations with Rb1, Rh1, Rc, Rb2, Rd, and Rg3. In contrast, the expression of *PgNLP07* showed significant positive correlated with all eight ginsenosides, with absolute correlation coefficients ranging from 0.355 to 0.546 (**Figure 10**). Although the previously screened *PgNLP02–02* gene exhibited a significant transcriptional response to MeJA elicitation, no statistically significant correlations were detected between this gene and any of the eight monomeric ginsenosides in the correlation analysis. For this reason, this gene was not presented in the final results. These findings further validate the hypothesis that the *PgNLP* family contains functionally divergent genes with opposite regulatory directions for the same biological process. In the phylogenetic analysis, *PgNLP02–01* and *PgNLP07* clustered within the same clade. These two sets of results mutually confirmed that these two genes likely function as crucial members involved in the responses to plant hormones and abiotic stress in *Panax ginseng*.

### 3.11 Expression pattern analysis of *PgNLP* and key enzyme genes under MeJA treatment

**Figure 11:**
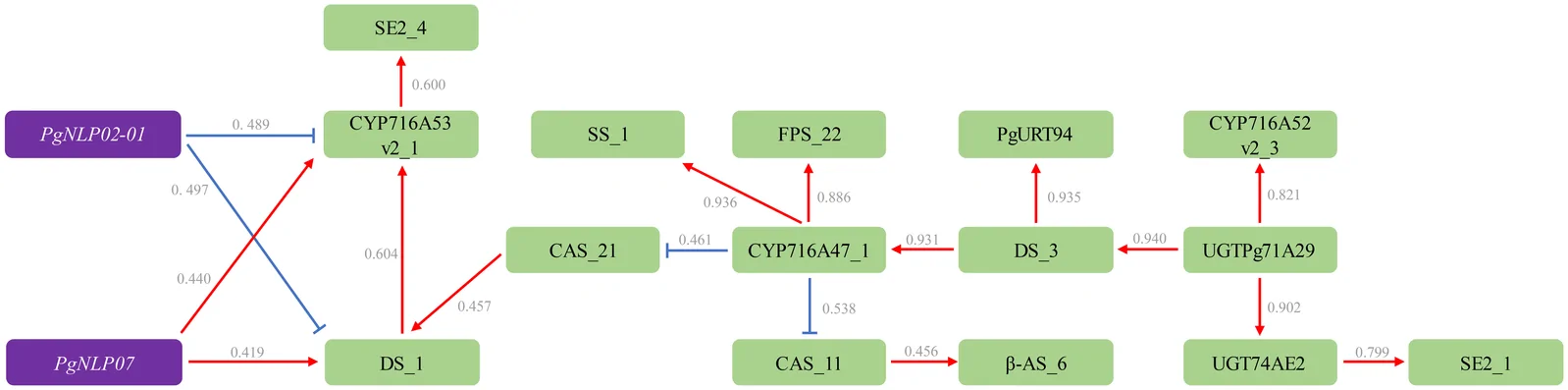
Putative regulatory network of *PgNLP02-01* and *PgNLP07* in regulating ginsenoside biosynthesis. Purple rounded rectangles represent *PgNLP* genes that are significantly correlated with ginsenosides under MeJA regulation, and green rounded rectangles represent key enzymes. Red arrows and blue arrows indicate positive and negative correlations at p ≤ 0.05, respectively.

Correlation analysis was performed on the expression levels of the three candidate genes and 15 key enzyme-encoding genes after MeJA treatment. These results indicate that *PgNLP02–01* and *PgNLP07* regulate ginsenoside biosynthesis by modulating the expression of *CYP716A53v2_1* and *DS_1* (**Figure 11**). *CYP716A53v2_1* catalyzes the conversion of protopanaxadiol to protopanaxatriol, whereas *DS_1* catalyzes the synthesis of dammarendiol-II from 2,3-oxidosqualene; both enzymes are key rate-limiting enzymes in the ginsenoside biosynthesis pathway. Under methyl jasmonate treatment, *PgNLP02–01* showed negative correlations with the expression of the two key enzymes and the contents of seven ginsenosides, whereas *PgNLP07* exhibited positive correlations with all nine ginsenosides and the expression levels of the two key enzymes. This consistent correlation pattern further supports the previous speculation.

## 4 Discussion

In this study, 20 members of the *NLP* gene family were identified in *Panax ginseng* via gene homology comparison, protein structure analysis, and conserved domain characterization. These 20 transcripts were alternatively spliced from only six coding genes, indicating that the differentiation and expansion of the *NLP* gene family during ginseng evolution did not primarily depend on tandem or whole-genome duplication. Instead, functional diversification occurs through non-homologous chromosome crossover and post-transcriptional alternative splicing (Wang et al., 2013; Capovilla et al., 2017; Liu et al., 2025). This evolutionary feature is supported by our experimental results. Although *PgNLP03* produced eight transcripts and *PgNLP05* generated six transcripts, Markov clustering based on expression profiles across 42 ginseng landraces showed that their transcripts did not cluster into a single clade, and some transcripts exhibited divergent-expression patterns. Tissue expression analysis of *PgNLP* genes across 14 tissue types revealed that *PgNLP03–03* and *PgNLP03–05* were expressed in all examined tissues. In contrast, *PgNLP03–08* showed weak expression specifically in fruit pedicels and was undetectable in other tissues. These results suggest that *NLP* genes in ginseng accomplish distinct biological functions mainly through alternative splicing (Lam et al., 2022).

In the final screening of *PgNLP* genes that were highly correlated with ginsenoside biosynthesis, three-dimensional association analyses integrating gene SNP/InDel variations, gene expression levels, and phenotypic data were adopted in this study. This screening strategy improved the efficiency and accuracy of candidate gene identification. Genome-wide association studies (GWAS) utilize SNPs and InDels as molecular genetic markers to identify potential functional loci based on phenotypic variation. Currently, SNP/InDel data have been widely applied in the identification of genes regulating quantitative traits (Yang et al., 2025; Zhou et al., 2025), genes associated with growth and development (Wang et al., 2020; López-Ruíz et al., 2024), and stress tolerance genes (Nyasulu et al., 2024; Wang et al., 2025). The correlation between gene expression and phenotypic traits has long been employed for gene mining, and the fundamental principle of weighted gene co-expression network analysis (WGCNA) relies heavily on the association analysis between gene expression and phenotypic characteristics. In this study, three complementary strategies were combined: association analysis of *PgNLP* SNPs/InDels with ginsenoside content, correlation analysis between *PgNLP* expression and ginsenoside accumulation, and co-expression network analysis of *PgNLP* with key enzyme genes involved in the ginsenoside biosynthesis pathway. Candidate genes screened from these three independent perspectives were supported by solid evidence at three levels: molecular genetic variation, transcriptional expression patterns, and co-expression within the biosynthetic pathway. This integrative approach effectively avoided false-positive results and one-sided limitations caused by a single screening method.

Ginseng is an allotetraploid species with a relatively high heterozygosity level, and pure inbred lines are currently unavailable for research (Jang et al., 2020). Therefore, all 346 sequencing samples used in this study were natural ginseng germplasm. These accessions were originally collected from 14 major ginseng-producing counties across four regions in Jilin Province, China, and subsequently cultivated at a dedicated ginseng experimental station to eliminate environmental effects on phenotypic traits. These samples represent the germplasm composition and molecular genetic variation diversity of ginseng resources in Jilin Province. Both *PgNLP02–01* and *PgNLP07* exhibited significant effects in the association analysis between SNP/InDel variations and ginsenoside content. Moreover, these genetic loci exerted markedly stronger influences on protopanaxadiol-type ginsenosides than on protopanaxatriol-type ginsenosides, suggesting that these two genes preferentially regulate the biosynthesis of dammarane-type protopanaxadiol ginsenosides under normal growth conditions. Furthermore, after verifying the expression patterns of *PgNLP* genes under MeJA treatment, a correlation analysis was performed between their relative expression levels and ginsenoside content. This analysis fully linked MeJA induction, ginsenoside accumulation, and *PgNLP* gene expression, providing direct statistical evidence that *PgNLP* genes exert regulatory effects on ginsenoside biosynthesis following MeJA treatment. To explore the underlying regulatory mechanism, a putative interaction network was constructed based on the correlation between the expression of *PgNLP* genes and key enzyme genes following MeJA treatment. These findings suggest that *PgNLP02–01* and *PgNLP07* modulate ginsenoside biosynthesis by regulating the expression of key enzyme genes, *CYP716A53v2_1* and *DS_1*. Nevertheless, limitations still existed in the present study. The mechanistic experiments were not conducted to elucidate how the two target genes modulate the expression of key enzyme genes and thereby ultimately affect MeJA-induced ginsenoside biosynthesis. The current research was merely confined to the correlation analysis between gene expression levels and ginsenoside contents, which will serve as an important direction for further in-depth mechanistic investigation in future work. These findings provide a theoretical reference and directional guidance for further in-depth investigation of the molecular mechanisms by which *PgNLP* genes regulate ginsenoside biosynthesis in ginseng.

## Data Availability

The datasets presented in this study can be found in online repositories. The names of the repository/repositories and accession number(s) can be found in the article/**Supplementary Material**.

## References

[B1] Bai Y. Wang J. Tang W. Sun D. Wang S. Chen K. . (2024). Genome-wide identification of NLP gene families and haplotype analysis of SiNLP2 in foxtail millet (setaria italica). Int. J. Mol. Sci. 25, 12938. doi: 10.3390/ijms252312938 39684649 PMC11641658

[B2] Bowman J. L. Kohchi T. Yamato K. T. Jenkins J. Shu S. Ishizaki K. . (2017). Insights into land plant evolution garnered from the marchantia polymorpha genome. Cell 171, 287–304. doi: 10.1016/j.cell.2017.09.030 28985561

[B3] Capovilla G. Symeonidi E. Wu R. Schmid M. (2017). Contribution of major FLM isoforms to temperature-dependent flowering in arabidopsis thaliana. J. Exp. Bot. 68, 5117–5127. doi: 10.1093/jxb/erx328 29036339 PMC5853260

[B4] Chardin C. Girin T. Roudier F. Meyer C. Krapp A. (2014). The plant RWP-RK transcription factors: key regulators of nitrogen responses and of gametophyte development. J. Exp. Bot. 65, 5577–5587. doi: 10.1093/jxb/eru261 24987011

[B5] De Smet R. Adams K. L. Vandepoele K. Van Montagu M. C. E. Maere S. Van de Peer Y. (2013). Convergent gene loss following gene and genome duplications creates single-copy families in flowering plants. Proc. Natl. Acad. Sci. U.S.A. 110, 2898–2903. doi: 10.1073/pnas.1300127110 23382190 PMC3581894

[B6] Ding Y. Yang H. Wu S. Fu D. Li M. Gong Z. . (2022). CPK28-NLP7 module integrates cold-induced ca^2+^ signal and transcriptional reprogramming in arabidopsis. Sci. Adv. 8, eabn7901. doi: 10.1126/sciadv.abn7901 35767615 PMC9242591

[B7] Han J. Y. Jo H. J. Choi Y. E. (2020). Overexpression of the squalene epoxidase gene (PgSE1) resulted in enhanced production of ginsenosides and phytosterols in transgenic ginseng. Plant Biotechnol. Rep. 14, 673–682. doi: 10.1007/s11816-020-00643-4 30311153

[B8] Hu S. Y. (1976). The genuspanax (ginseng) in chinese medicine. Econ. Bot. 30, 11–28. doi: 10.1007/BF02866780 30311153

[B9] Jang J. He Z. Huang L. Hwang J. Y. Kim M. Y. Cho J. Y. (2024). Upregulation of NK cell activity, cytokine expression, and NF-κb pathway by ginsenoside concentrates from panax ginseng berries in healthy mice and macrophage cell lines. J. Ethnopharmacol. 335, 118681. doi: 10.1016/j.jep.2024.118681 39121929

[B10] Jang W. Jang Y. Kim N. H. Waminal N. E. Kim Y. C. Lee J. W. . (2020). Genetic diversity among cultivated and wild panax ginseng populations revealed by high-resolution microsatellite markers. J. Ginseng Res. 44, 637–643. doi: 10.1016/j.jgr.2019.05.008 32617044 PMC7322750

[B11] Jiang Y. Zhang Q. Zeng Z. Wang Y. Zhao M. Wang K. . (2024). The AP2/ERF transcription factor PgERF120 regulates ginsenoside biosynthesis in ginseng. Biomolecules 14, 345. doi: 10.3390/biom14030345 38540764 PMC10968211

[B12] Jiang T. Zhang Y. Zuo G. Luo T. Wang H. Zhang R. . (2024). Transcription factor PgNAC72 activates DAMMARENEDIOL SYNTHASE expression to promote ginseng saponin biosynthesis. Plant Physiol. 195, 2952–2969. doi: 10.1093/plphys/kiae202 38606940

[B13] Kim Y. S. Hahn E. J. Murthy H. N. Paek K. E. Y. (2004). Adventitious root growth and ginsenoside accumulation in panax ginseng cultures as affected by methyl jasmonate. Biotechnol. Lett. 26, 1619–1622. doi: 10.1007/s10529-004-3183-2 15604808

[B14] Kim T. E. D. Han J. Y. Huh G. H. Choi Y. E. (2011). Expression and functional characterization of three squalene synthase genes associated with saponin biosynthesis in panax ginseng. Plant Cell Physiol. 52, 125–137. doi: 10.1093/pcp/pcq179 21134898

[B15] Kim Y. K. Kim Y. B. Uddin M. R. Lee S. Kim S. U. Park S. U. (2014). Enhanced triterpene accumulation in panax ginseng hairy roots overexpressing mevalonate-5-pyrophosphate decarboxylase and farnesyl pyrophosphate synthase. ACS Synth. Biol. 3, 773–779. doi: 10.1021/sb400194g 24933610

[B16] Konishi M. Yanagisawa S. (2013). Arabidopsis NIN-like transcription factors have a central role in nitrate signalling. Nat. Commun. 4, 1617. doi: 10.1038/ncomms2621 23511481

[B17] Konishi M. Yanagisawa S. (2019). The role of protein-protein interactions mediated by the PB1 domain of NLP transcription factors in nitrate-inducible gene expression. BMC Plant Biol. 19, 90. doi: 10.1186/s12870-019-1692-3 30819094 PMC6393987

[B18] Lam P. Y. Wang L. Lo C. Zhu F. Y. (2022). Alternative splicing and its roles in plant metabolism. Int. J. Mol. Sci. 23, 7355. doi: 10.3390/ijms23137355 35806361 PMC9266299

[B19] Lee M. H. Jeong J. H. Seo J. W. Shin C. G. Kim Y. S. In J. G. . (2004). Enhanced triterpene and phytosterol biosynthesis in panax ginseng overexpressing squalene synthase gene. Plant Cell Physiol. 45, 976–984. doi: 10.1093/pcp/pch126 15356323

[B20] Li L. Wang K. Zhao M. Li S. Jiang Y. Zhu L. . (2019). Selection and validation of reference genes desirable for gene expression analysis by qRT-PCR in MeJA-treated ginseng hairy roots. PloS One 14, e0226168. doi: 10.1371/journal.pone.0226168 31805178 PMC6894815

[B21] Li L. Wang Y. Zhao M. Wang K. Sun C. Zhu L. . (2021). Integrative transcriptome analysis identifies new oxidosqualene cyclase genes involved in ginsenoside biosynthesis in jilin ginseng. Genomics 113, 2304–2316. doi: 10.1016/j.ygeno.2021.05.023 34048908

[B22] Lian J. Han H. Chen X. Chen Q. Zhao J. Li C. (2022). Stemphylium lycopersici nep1-like protein (NLP) is a key virulence factor in tomato gray leaf spot disease. J. Fungi 8, 518. doi: 10.3390/jof8050518 35628773 PMC9144795

[B23] Liu M. Chang W. Fan Y. Sun W. Qu C. Zhang K. . (2018). Genome-wide identification and characterization of NODULE-INCEPTION-like protein (NLP) family genes in brassica napus. Int. J. Mol. Sci. 19, 2270. doi: 10.3390/ijms19082270 30072649 PMC6121332

[B24] Liu S. Jiang Y. Wang Y. Huo H. Cilkiz M. Chen P. . (2023). Genetic and molecular dissection of ginseng (panax ginseng mey.) Germplasm using high-density genic SNP markers, secondary metabolites, and gene expressions. Front. Plant Sci. 14, 1165349. doi: 10.3389/fpls.2023.1165349 37575919 PMC10416250

[B25] Liu K. H. Liu M. Lin Z. Wang Z. F. Chen B. Liu C. . (2022). NIN-like protein 7 transcription factor is a plant nitrate sensor. Sci. (New York N.Y.) 377, 1419–1425. doi: 10.1126/science.add1104 36137053 PMC9628810

[B26] Liu R. Zhao D. Li P. Xia D. Feng Q. Wang L. . (2025). Natural variation in OsMADS1 transcript splicing affects rice grain thickness and quality by influencing monosaccharide loading to the endosperm. Plant Commun. 6, 101178. doi: 10.1016/j.xplc.2024.101178 39489992 PMC11783882

[B27] Long X. Qiu C. Sun J. Song T. Li J. Jin H. . (2026). Identification of the NLP gene family in populus euphratica and its expression analysis under drought stress. Int. J. Mol. Sci. 27, 3071. doi: 10.3390/ijms27073071 41977257 PMC13073922

[B28] López-Ruíz B. A. García-Ponce B. de la Paz Sánchez M. Álvarez-Buylla E. R. Urrutia A. O. Garay-Arroyo A. (2024). Genome-wide association studies meta-analysis uncovers NOJO and SGS3 novel genes involved in arabidopsis thaliana primary root development and plasticity. Mol. Biol. Rep. 51, 763. doi: 10.1007/s11033-024-09623-1 38874813 PMC11178574

[B29] Lyu F. Han F. Ge C. Mao W. Chen L. Hu H. . (2023). OmicStudio: a composable bioinformatics cloud platform with real-time feedback that can generate high-quality graphs for publication. iMeta 2, e85. doi: 10.1002/imt2.85 38868333 PMC10989813

[B30] Mishra S. Singroha G. Tiwari R. Sharma P. (2025). Comprehensive genome-wide expression analysis of NLP transcription factors elucidates their crucial role in enhancing nitrogen response in wheat (triticum aestivum l.). Curr. Plant Biol. 42, 100463. doi: 10.1016/j.cpb.2025.100463 38826717

[B31] Ning M. Li Q. Wang Y. Li Q. Tao Y. Zhang F. . (2025). Alternative splicing drives the functional diversification of a bHLH transcription factor in the control of growth and drought tolerance in rice. Sci. Bull. 70, 153–156. doi: 10.1016/j.scib.2024.06.001 38880686

[B32] Nyasulu M. Zhong Q. Li X. Liu X. Wang Z. Chen L. . (2024). Uncovering novel genes for drought stress in rice at germination stage using genome wide association study. Front. Plant Sci. 15, 1421267. doi: 10.3389/fpls.2024.1421267 39148613 PMC11325455

[B33] Ru W. Wang D. Xu Y. He X. Sun Y. E. Qian L. . (2015). Chemical constituents and bioactivities of panax ginseng (c. A. Mey.). Drug Discov. Ther. 9, 23–32. doi: 10.5582/ddt.2015.01004 25788049

[B34] Sato T. Maekawa S. Konishi M. Yoshioka N. Sasaki Y. Maeda H. . (2017). Direct transcriptional activation of BT genes by NLP transcription factors is a key component of the nitrate response in arabidopsis. Biochem. Biophys. Res. Commun. 483, 380–386. doi: 10.1016/j.bbrc.2016.12.135 28025145

[B35] Schauser L. Roussis A. Stiller J. Stougaard J. (1999). A plant regulator controlling development of symbiotic root nodules. Nature 402, 191–195. doi: 10.1038/46058 10647012

[B36] Schauser L. Wieloch W. Stougaard J. (2005). Evolution of NIN-like proteins in arabidopsis, rice, and lotus japonicus. J. Mol. Evol. 60, 229–237. doi: 10.1007/s00239-004-0144-2 15785851

[B37] Wang D. Liu X. He G. Wang K. Li Y. Guan H. . (2025). GWAS and transcriptome analyses unravel ZmGRAS15 regulates drought tolerance and root elongation in maize. BMC Genomics 26, 246. doi: 10.1186/s12864-025-11435-x 40082805 PMC11907892

[B38] Wang T. Wei L. Wang J. Xie L. Li Y. Y. Ran S. . (2020). Integrating GWAS, linkage mapping and gene expression analyses reveals the genetic control of growth period traits in rapeseed (brassica napus l.). Biotechnol. Biofuels 13, 134. doi: 10.1186/s13068-020-01774-0 32774455 PMC7397576

[B39] Wang Z. Zhang L. Sun C. Gu R. Mi G. Yuan L. (2018). Phylogenetic, expression and functional characterizations of the maize NLP transcription factor family reveal a role in nitrate assimilation and signaling. Physiol. Plant. 163, 10–111. doi: 10.1111/ppl.12696 29364528

[B40] Wang C. Zhu H. Jin L. Chen T. Wang L. Kang H. . (2013). Splice variants of the SIP1 transcripts play a role in nodule organogenesis in lotus japonicus. Plant Mol. Biol. 82, 97–111. doi: 10.1007/s11103-013-0042-3 23494209

[B41] Xie J. Chen Y. Cai G. Cai R. Hu Z. Wang H. (2023). Tree visualization by one table (tvBOT): a web application for visualizing, modifying and annotating phylogenetic trees. Nucleic Acids Res. 51, W587–W592. doi: 10.1093/nar/gkad359 37144476 PMC10320113

[B42] Xin T. Zhang Z. Zhang Y. Li X. Wang S. Wang G. . (2025). Recessive epistasis of a synonymous mutation confers cucumber domestication through epitranscriptomic regulation. Cell 188, 4517–4529. doi: 10.1016/j.cell.2025.06.007 40602402

[B43] Xin W. Zheng H. Yang L. Xie S. Xia S. Wang J. . (2025). Genome-wide association studies identify OsNLP6 as a key regulator of nitrogen use efficiency in rice. Plant Biotechnol. J. 23, 5110–5125. doi: 10.1111/pbi.70296 40755248 PMC12576451

[B44] Yan D. Easwaran V. Chau V. Okamoto M. Ierullo M. Kimura M. . (2016). NIN-like protein 8 is a master regulator of nitrate-promoted seed germination in arabidopsis. Nat. Commun. 7, 13179. doi: 10.1038/ncomms13179 27731416 PMC5064020

[B45] Yang L. He W. Zhu Y. Lv Y. Li Y. Zhang Q. . (2025). GWAS meta-analysis using a graph-based pan-genome enhanced gene mining efficiency for agronomic traits in rice. Nat. Commun. 16, 3171. doi: 10.1038/s41467-025-58081-1 40180959 PMC11968974

[B46] Ye K. Bu F. Zhong L. Dong Z. Ma Z. Tang Z. . (2024). Mapping the molecular landscape of lotus japonicus nodule organogenesis through spatiotemporal transcriptomics. Nat. Commun. 15, 6387. doi: 10.1038/s41467-024-50737-8 39080318 PMC11289483

[B47] Yu J. Xuan W. Tian Y. Fan L. Sun J. Tang W. . (2021). Enhanced OsNLP4-OsNiR cascade confers nitrogen use efficiency by promoting tiller number in rice. Plant Biotechnol. J. 19, 167–176. doi: 10.1111/pbi.13450 32710800 PMC7769241

[B48] Zhou Y. Heng Y. Chen S. Wang J. He K. Geng J. . (2025). Dissecting the genetic basis of agronomic traits by multi-trait GWAS and genetic networks in maize (zea mays l.). aBIOTECH 6, 707–725. doi: 10.1007/s42994-025-00241-4 41312106 PMC12647461

